# Up-Flow Anaerobic Sludge Blanket (UASB) Technology for Energy Recovery: A Review on State-of-the-Art and Recent Technological Advances

**DOI:** 10.3390/bioengineering7020043

**Published:** 2020-05-10

**Authors:** Matia Mainardis, Marco Buttazzoni, Daniele Goi

**Affiliations:** Department Polytechnic of Engineering and Architecture (DPIA), University of Udine, Via del Cotonificio 108, 33100 Udine, Italy; buttazzoni.marco.1@spes.uniud.it (M.B.); daniele.goi@uniud.it (D.G.)

**Keywords:** UASB, co-digestion, biogas, high-rate anaerobic digestion, energy recovery, granular sludge, renewable energy, decentralized wastewater treatment, two-stage anaerobic digestion, Anammox

## Abstract

Up-flow anaerobic sludge blanket (UASB) reactor belongs to high-rate systems, able to perform anaerobic reaction at reduced hydraulic retention time, if compared to traditional digesters. In this review, the most recent advances in UASB reactor applications are critically summarized and discussed, with outline on the most critical aspects for further possible future developments. Beside traditional anaerobic treatment of soluble and biodegradable substrates, research is actually focusing on the treatment of refractory and slowly degradable matrices, thanks to an improved understanding of microbial community composition and reactor hydrodynamics, together with utilization of powerful modeling tools. Innovative approaches include the use of UASB reactor for nitrogen removal, as well as for hydrogen and volatile fatty acid production. Co-digestion of complementary substrates available in the same territory is being extensively studied to increase biogas yield and provide smooth continuous operations in a circular economy perspective. Particular importance is being given to decentralized treatment, able to provide electricity and heat to local users with possible integration with other renewable energies. Proper pre-treatment application increases biogas yield, while a successive post-treatment is needed to meet required effluent standards, also from a toxicological perspective. An increased full-scale application of UASB technology is desirable to achieve circular economy and sustainability scopes, with efficient biogas exploitation, fulfilling renewable energy targets and green-house gases emission reduction, in particular in tropical countries, where limited reactor heating is required.

## 1. Introduction

Nowadays, the over dependence on fossil fuels poses global risks, such as resources depletion and increasing climate change, due to the net increase in CO_2_ levels in the atmosphere [1]. Anaerobic digestion (AD) is one of the most promising technologies, breaking complex organic substrates into biogas [2] that is substantially composed of a mixture of methane and carbon dioxide. AD, being 100% renewable, is an effective and environmental-friendly waste and wastewater management technique and can be considered as one of the most important renewable energy sources, due to CH_4_ generation during the digestion process [3]. However, biogas generation from different streams, together with utilization in energy applications, is still somewhat challenging due to complex waste physicochemical properties, affecting biomass metabolic pathways and methane yield [2]. 

AD requires less energy than other thermochemical methods, such as gasification and pyrolysis, due to the low operating temperature [4], and consequently AD application throughout the world has continuously increased in the last decades. Beside highly biodegradable streams, advances in research allowed to apply AD also to lignocellulosic substrates, characterized by slow hydrolysis kinetics, such as macroalgal biomass [5], switchgrass [6] and yard waste [7], widening the spectrum of suitable matrices for biogas production. Proper pre-treatment application before AD or creating a mixture of complementary substrates can significantly increase process efficiency and consequently biogas yield [4]. Apart from large-scale plants, AD can be applied also to small-to-medium enterprises (SMEs), contributing to local energy and environmental sustainability, if the produced organic substrates have a suitable methane potential (typically evaluated by means of Biochemical Methane Potential (BMP) tests) [8]. An increased biogas production helps to augment renewable energy production and penetration in the fossil fuel market, as sustained by European Union (EU) sustainable development programs [9]. 

High-rate anaerobic digesters, in particular, received great attention in recent years, due to their high loading capacity and low sludge production [10]. High-rate reactors, by uncoupling biomass retention (expressed as solid retention time, SRT) and liquid retention (hydraulic retention time, HRT), allow to significantly reduce the required reactor volume, if compared to traditional systems [11]. Among this wide category, up-flow anaerobic sludge blanket (UASB) reactor is the most widely applied system worldwide [10]. UASB reactor was developed in the 1970s in the Netherlands and its application rapidly increased, due to its excellent reported performances on different biodegradable wastewater streams [12]. The key feature of a UASB reactor is the granular sludge, that retains highly active biomass with excellent settling abilities in the reactor [11], showing a very low sludge volume index (SVI), consequently improving also sludge-effluent separation. 

**Figure 1:**
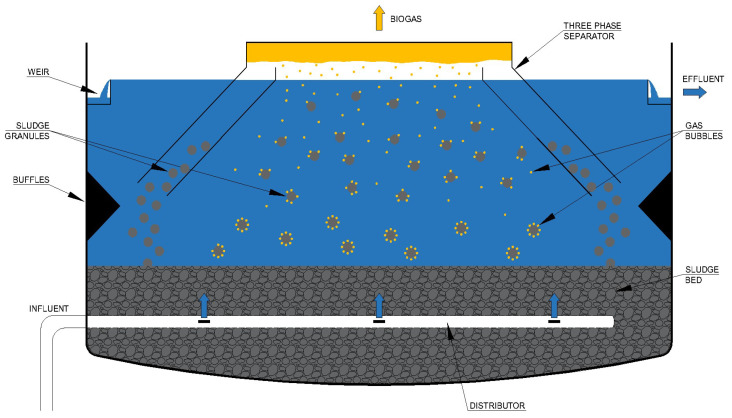
Up-flow anaerobic sludge blanket (UASB) reactor process scheme.

A simplified scheme of a UASB reactor is reported in Figure 1. Wastewater enters at the bottom of the reactor and flows upwards through a so-called “sludge blanket”, consisting of a granular sludge bed [13]. UASB configuration enables an extremely efficient mixing between the biomass and the wastewater, leading to a rapid anaerobic decomposition [13]. The operation of a UASB reactor fundamentally revolves on its granular sludge bed, that gets expanded as the wastewater is made to flow vertically upwards through it [14]. The microflora attached to the sludge particles removes the pollutants contained in wastewater, thus biofilm quality and the intimacy of sludge-wastewater contact are among the key factors governing UASB reactor success [14]. The generated biogas facilitates the mixing and the contact between sludge and wastewater, and the three phase gas-liquid-solid separator, located in the upper part of the reactor, allows to extract biogas, separating it from liquid effluent and residual sludge particles [13]. Typical geometrical and operating characteristics of UASB reactor include a height to diameter ratio of 0.2–0.5 and an up-flow velocity of 0.5–1.0 m/h [12]. 

UASB treatment, if compared to aerobic stabilization, requires lower energy consumption, is efficient at higher loading rate and needs limited micro and macro-nutrients, producing a reduced amount of sludge, that is characterized by an improved dewaterability [12]. In fact, only about 5–10% of the organic matter in wastewater is transferred to the sludge fraction [12]. On the other hand, UASB treatment is known to have a limited effect on nutrients (nitrogen and phosphorous), as well as on micro-pollutants [15]. UASB treatment of high-strength industrial wastewater allows to significantly reduce energy expenses for aeration in wastewater treatment plants (WWTP), if UASB is applied as a pre-treatment before secondary biological process [16]. UASB reactor is able to efficiently treat various high-strength industrial wastewater (such as brewery wastewater [17], sugarcane vinasse [18], paper mill wastewater [16], dairy wastewater [16]), characterized by high chemical oxygen demand (COD) concentration and substantial biodegradability (high biochemical oxygen demand (BOD)/COD ratio). UASB treatment of high-loaded substrates allows to get high methane yields with a reduced energy requirement (if compared to aerobic stabilization) and a significantly lower excess sludge production [19]. Furthermore, recently UASB has proved to be efficient also on diluted streams, such as municipal wastewater [12], even at ambient operating temperature.

UASB treatment was compared to aerobic open lagoon in the work reported in [20] to treat wastewater from ethanol production, highlighting that the environmental cost of open lagoon is greater than UASB reactor. UASB reactor and anaerobic membrane bioreactor (AnMBR) are particularly indicated for treating chemical-industrial wastewater [21]. However, a further exploitation of anaerobic treatment is actually curbed by the ineffective mineralization of degradation-resistant organic substrates [21], thus a proper pre-treatment needs to be applied to enhance anaerobic degradability.

The most important aspects to be controlled when applying a UASB treatment are reactor start-up and granulation enhancement, coupling the anaerobic section with a post-treatment unit to efficiently abate organic matter, nutrients and pathogens [10]. A sufficient inoculation must be provided at the beginning of operations to reduce drawbacks such as process sensitivity, vulnerability, odor emission, long start-up period [12]. UASB operation start-up typically requires that 10–30% of the volume is inoculated with active granular biomass [12]. Given that the long start-up and the slow granulation are major constraints (in particular when treating complex and refractory wastewater), recently it was proved that granulation could be stimulated by chemical addition, such as calcium sulphate, enhancing granulation rate and improving methanogenic activity [22]. An increased granule formation (>0.25 mm) in the range of 7–40% was reported in a UASB reactor with CaSO_4_ addition after 90 d in comparison with control, with an increased COD removal efficiency (3–9%) at moderate organic loading rate (OLR) (2.89 kg COD/m^3^d) [22]. Granular biomass is able to tolerate higher hazardous and toxic compound concentration than traditional flocculent sludge: as an example, UASB was shown to tolerate higher organic loading rate (OLR) than anaerobic membrane bioreactor (AnMBR) when treating N, N-dimethylformamide [23].

In this review, the most recent advances in UASB anaerobic treatment are presented and discussed, with a focus on the most critical aspects for possible further developments. In Section 2, the most recent literature results regarding methane yields and operating parameters from a variety of substrates are presented, while in Section 3 the advances in UASB hydrodynamic understanding and microbial community composition are discussed. In Section 4, the most recent outcomes regarding two-stage UASB digestion are highlighted, while in Section 5 co-digestion applied to high-rate anaerobic treatment is presented. In Section 6, UASB application as Anammox process is introduced. Modified UASB systems are successively presented in Section 7. A particular focus on low-temperature decentralized UASB treatment of municipal streams is done in Section 8. Section 9 specifically deals with pre-treatments before AD and post-treatment of the treated anaerobic effluent with a plant-wide perspective. Section 10 analyzes toxicity aspects in UASB treatment, considering meaningful recent literature outcomes. The most critical aspects and future perspectives, with a focus on the energy and environmental aspects, are finally discussed in Section 11. 

The main aim of the paper is to provide an up-to-date vision of the engineering aspects of UASB reactor application, together with forecasting possible further advancements. Most of the literature studies provide the results of laboratory and pilot-tests, while full-scale applications are still limited, in particular on slowly degradable substrates. An increased exploitation of biogas generation from liquid and solid substrates is strongly recommended to increase renewable energy share in the global market: high-rate anaerobic reactors can help to move towards an increased sustainable energy generation, in particular in tropical and decentralized areas.

## 2. UASB Reactor: Substrate Characteristics and Operating Conditions

In Section 2.1, the most recent literature outcomes regarding UASB treatment are critically summarized, while in Section 2.2 the influence of operating conditions is discussed; in Section 2.3, advanced high-rate reactors, developed from original UASB, are presented. 

### 2.1. Substrate Characteristics

Substrate characteristics play a major role in UASB process efficiency: a high nitrogen concentration and a significant particulate matter content in the influent wastewater can lead to an excessive ammonia accumulation, which is notoriously toxic (after a certain threshold), and to a slow hydrolysis phase, reducing biogas production rate [24]. Some of the most recently reported literature outcomes regarding UASB treatment of different high-loaded substrates were summarized in Table 1. Besides traditional highly biodegradable substrates, research is actually focusing on refractory (chemical or industrial wastewater) and diluted (municipal wastewater) streams to extend the applicability of high-rate anaerobic reactors. Typically, complex wastewater requires a two-stage digestion (Section 4) or the selection of a proper pre-treatment (Section 9) to increase its biodegradability. UASB treatment of municipal wastewater, instead, is difficult to apply due to diluted stream characteristics, and is specifically described in Section 8. UASB reactor has shown to be particularly effective in degrading highly biodegradable substrates, where short HRTs (<24 h) can be applied, together with consistent OLRs (>20 kg COD/m^3^d), obtaining high COD removal efficiency (>90%) (Table 1). 

Most of the studies regarding UASB treatment report mesophilic operations, which proved to be particularly effective in coupling a high biogas yield and a good process stability. A great variability in the applied operating conditions emerges from Table 1, both in terms of OLR and HRT, demonstrating that a substrate-specific approach is required to obtain a high COD abatement and a satisfactory methane yield. The highest abatements arise when treating highly biodegradable and soluble components, where the particulate fraction (difficult to hydrolyze) is limited. The treatment of extremely loaded substrates (COD > 100 g/L) leads to the necessity of adopting longer HRT, to have an efficient treatment, while streams characterized by lower COD concentration (<2 g/L) generally produce a lower methane yield, due to the reduced OLR. Moreover, it appears from Table 1 that standardization of methane yields from literature results is complex, due to the different adopted unity of measure. 

Salinity is an important parameter in UASB anaerobic treatment when treating brackish streams: recent research proved that a high substrate removal can be achieved even under a salinity level of 10 g NaCl/L [29]. However, lower salt conditions stimulate the formation of larger granules and a faster degradation rate [29]. Moreover, it was seen that salinity does not substantially modify microbial community composition, even if methanogen abundance is reduced [29]. In a consistent way, a previous study on phenol UASB treatment demonstrated that the granular biomass is able to tolerate moderate salinity levels (up to 10 g Na^+^/L), while higher salt levels (10–20 g Na^+^/L) reduce reactor efficiency [37].

The treatment of substrates available in a specific territory is fundamental to achieve local circular economy and sustainability visions. Waste and wastewater can be valorized on-site to produce electric energy, fulfilling a high share of plant total need, and heat (that can be used for district heating). As an example, pistachio wastewater was tested as a possible feed for UASB reactor in the work in [31] and a potential to produce up to 28,200 MWh of electric energy from biogas was highlighted in Turkey by considering annual wastewater production (520,000 m^3^/y) [31]. 

Wastewater containing high lipid concentration is particularly critical to be treated, given to a number of drawbacks such as clogging, sludge floatation, formation of foams and odor emission, biomass washout. However, lipid-rich wastewater has a higher methane potential (0.99 L CH_4_/g) than proteins (0.63 L CH_4_/g) and carbohydrates (0.42 L CH_4_/g) [38]. Consistently, the treatment of slaughterhouse wastewater in a UASB reactor has to face with the inability to operate at high OLRs, as a result of suspended and colloidal impurities, including cellulose, proteins and fats, abundantly present in this stream [39]. In these situations, in order to achieve the desired efficiency, UASB reactor must be often coupled with an efficient post-treatment. In addition, substrate pre-treatment can be beneficial for increasing its biodegradability and the subsequent obtainable methane yield. The available pre- and post-treatment technologies are specifically described in Section 9. As an example, a semi-continuous process for slaughterhouse wastewater treatment was proposed in the work in [33], followed by a photoelectro-Fenton (PEF) treatment [33]. Wastewater from fish processing industry, instead, was studied in the work in [40] as another lipid-rich wastewater stream, suggesting a complex treatment scheme (baffled moving bed biofilm reactor followed by UASB reactor, fluidized immobilized cell carbon oxidation and chemoautotrophic activated carbon oxidation), with excellent COD, protein, lipid, oil and grease abatement [40]. 

Finally, particular attention has to be given to wastewater having significant sulfur concentration (such as sugar cane vinasse), considering that the inner part of the UASB reactor is exposed to a higher H_2_S concentration than that measured in the treated effluent [41]. The influence of COD/SO_4_^2−^ ratio on starch wastewater biodegradation in a UASB reactor was studied in the work in [42] with a progressive COD/SO_4_^2−^ ratio decrease, highlighting a stable biogas production and a satisfactory COD and sulfate removal until COD/SO_4_^2−^ ≥ 2 [42]. A further decrease in COD/ SO_4_^2−^ ratio suppressed methanogenesis through electron competition and sulfide inhibition [42]. Crude glycerol was again investigated in [43], where a sulfidogenic UASB reactor was proposed, showing maximum COD removal efficiency at COD/S-sulfate ratio of 8.5 g O_2_/g S–SO_4_^2−^.

### 2.2. Influence of Operating Conditions

Beside substrate characteristics, the operational conditions play a major role in UASB process efficiency and stability. The main parameters that influence UASB performances are operating temperature (psychrophilic, mesophilic or thermophilic regime), pH, HRT, OLR, up-flow velocity. A stable pH, close to neutrality, is required to obtain a good-quality granular sludge, with sufficient alkalinity in the feeding substrate [14]. Up-flow velocity helps to maintain the mixing between sludge bed and wastewater, as well as to guarantee the desired HRT. The recommended up-flow velocity range in a UASB reactor is 0.5–1.5 m/h [11], even if values above 1 m/h in conventional UASB systems can lead to granule disintegration and biomass washout, due to shear stress that fragments the biomass [14]. A higher up-flow velocity is generally applied in the reactor start-up phase to select the biomass, removing smaller granules and maintaining the larger ones. As previously stated, the start-up of a UASB reactor is particularly critical and needs to be specifically controlled, with a progressive OLR increase. 

Regarding temperature, most of the reported literature studies include mesophilic operations, which are widely accepted to be a good compromise between a sufficient biomass activity and reactor stability. The transition of operating conditions between different temperature regimes is another noteworthy aspect to be investigated, due to instability effects that can arise. As an example, the shift from mesophilic to thermophilic temperature regime was studied in the work in [44], with glucose and ethanol as feed: a better resistance to temperature variations was observed using ethanol as substrate, finding a significant correlation between granular sludge conductivity and COD removal rate, as well as between *Geobacter* abundance and COD abatement [44]. An enhanced sludge conductivity means a frequent electron transfer, subsequently increasing the efficiency of methane production, mainly through direct interspecies electron transfer (DIET) mechanism [44]. The influence of temperature and particle dimensions on UASB treatment of swine manure slurry was analyzed in [45], highlighting that temperature effect was more pronounced on high-particle content substrate. In fact, while at 25 °C the methane yield from raw and centrifuged manure was comparable, at 35 °C a significantly higher methane production was obtained from raw manure, showing that small particles and soluble components were mainly digested at the lower tested range [45].

Regarding pH effect, a stable pH close to neutrality is optimal for continuous operations. A sudden pH reduction leads to an imbalance in anaerobic trophic chain, with accumulation of undegraded volatile fatty acid (VFA) and a further tendency to pH decrease, particularly if a limited alkalinity is present. Sugar refinery wastewater was treated in a UASB reactor in the work in [46] by reducing pH to 5.0 to analyze bacterial dynamics, showing that COD and methane yield reduced of about 25%, with a significant change in acidogenic biomass, leading to propionate increase and accumulation, together with block of metabolic balance [46]. In another study, it was showed that intermediate compounds from glycerol degradation are mainly propionic and acetic acid and detrimental effects on system performances (with acetic acid accumulation) were highlighted when UASB reactor was subjected to pH shocks [43]. 

Finally, as for organic load, a low starting OLR is typically required in start-up and transitory periods, to allow biomass adaption, followed by a moderate progressive increase towards final target value. An increasing bacterial specialization and a progressive decrease in methanogenic population was highlighted by step-wise augmenting OLR in glycerol UASB treatment, due to a functional biomass organization, that corresponded to a proportional increase in methane yield [47].

### 2.3. Advanced High-Rate Reactors

Continuous research on UASB reactor performances in the treatment of different substrates led to the development of advanced high-rate reactors, starting from the basic UASB configuration (Figure 1). These advanced systems include expanded granular sludge bed (EGSB) reactor, which allows an improved contact between granules and wastewater and an enhanced sludge separation (due to the rapid up-flow velocity), and static granular bed reactor (SGBR), which acts as an anaerobic filter, with absence of mixing and opposite gas and wastewater flows [48]. Internal Circulation (IC) reactor is another advanced high-rate system, based on a series connection of two UASB reactors: IC has a higher height/diameter ratio and shows proved advantages over conventional UASB, such as increased operating OLR, powerful stress resistance, operational stability, economic space utilization [49]. Recently, a high-rate External Circulation Sludge Bed (ECSB) reactor was proved to be beneficial for high-rate anaerobic treatment of cheese industry wastewater at full-scale, capable also to tolerate high influent calcium loads [50].

The most recent advances in high-rate AD include Internal Circulation Experience (ICX) systems, having a two-stage separation system that enables an excellent biomass retention [51]. Higher OLRs (up to 20–35 kg COD/m^3^d) can be applied in comparison to traditional UASB (10–15 kg COD/m^3^d) and IC (20–30 kg COD/m^3^d) reactors, maintaining at the same time a stable and high COD abatement [51]. The key feature of a ICX reactor is the two-stage phase separation, where the traditional three-phase separator is substituted by a top separator, that removes biogas from the reactor, and a bottom separator, that separates granular biomass from the treated effluent [51]. This system results in a higher biomass retention efficiency, if compared to UASB reactor [51]. More than 70 full-scale ICX reactors have been built since 2013, with a size ranging from 85 to 5000 m^3^, showing a high and stable COD removal even under fluctuating operating conditions [51].

## 3. UASB Hydrodynamics and Microbial Community

The deepening of UASB hydrodynamics is fundamental to understand and model UASB reactor, providing useful insights for process optimization. Hydrodynamic UASB modeling is complex and should integrate AD process dynamics (for example by applying the widely known anaerobic digestion model number 1 (ADM1), developed by International Water Association (IWA) [52]), flow pattern, biofilm characteristics and sulfate reduction process [53]. A non-ideal flow was shown to better simulate UASB hydrodynamics by evaluating fundamental hydrodynamic parameters, such as Peclet number and dispersion coefficient [53]. UASB reactor hydraulics can be described with a dispersive model or a multi-continuously stirred tank reactor (CSTR) model [54]. It was highlighted that a dispersive model simulates in a better way experimental data (if compared to the multi-CSTR approach), and could be integrated with the ADM1 bioprocess model to include both biological and hydrodynamic aspects [54]. A three-dimensional numerical hydrodynamic study was proposed in [55] to simulate a UASB reactor with different inclinations of the gas deflector, showing a good fitting between modelled and experimental data regarding flowrate, influent and effluent organic matter, solid concentration at liquid-gas interface, pressure field [55]. 

The core aspect of UASB technology is undoubtedly the granular sludge bed, which is identified by an extreme compactness and a high capability to tolerate harsh environments. The granules in UASB reactors are characterized by a layered structure, where hydrolytic and acidifying bacteria are located in the outer granule shell, while methanogens are positioned in the inner stratus [12]. Cavities and holes in the granules allow transporting gases, substrates and metabolites from a layer to other layers [12]. A granular sludge having proper particle size, density and microfilm characteristics enhance reactor efficiency [14]. The knowledge of microbial community composition is fundamental to optimize granule performances: it has been recently proved that reactor operating parameters are correlated with granule microstructure [56]. In particular, the loading ratio was identified as the key parameter controlling granule transformations [56]. Beside upward velocity, intermittent gas sparging could be an effective way to promote liquid shear, altering granule surface structure [56]. The detailed microscopical analysis of granule structure demonstrated that larger granules (3–4 mm diameter) have multi-layered internal microstructures, with more consistent methanogenic activity than smaller granules (1–2 mm diameter) [57]. Granular sludge was shown to have superior performances than thickened digestate and anaerobic sewage in UASB reactors [35].

The microbial community in UASB reactors is capable to adapt even to sudden changes in operating parameters that can arise in continuous operations. Bacteria response to alkaline pH perturbations in a full-scale UASB reactor treating dairy wastewater showed that after an accidental pH increase (which affected microbial community structure) the microbial population was able to regain diversity and methanogenic activity [58]. Granulation fundamentals are not completely understood at the present, despite the number of existing granulation-based treatment plants [59]. Anaerobic granulation substantially consists of a microbial aggregation where communities are organized into dense and highly-structured aggregates, not necessitating any carrier media [59]. Microbial community composition was shown to modify in continuous operations, depending on the treated substrate [60]. As an example, in the work in [60], it was observed that *Methanosaeta* and *Methanobacterium*, despite being absent in the original inoculum, became dominant archaea in phenol UASB treatment, while the dominant bacteria were *Syntrophorhabdus* (known to degrade phenol to benzoate and then benzoate to acetate and H_2_) and *Clostridium* [60].

A limit of the anaerobic trophic chain is the slow conversion metabolism of short-chain fatty acids and alcohols by syntrophic bacteria [61]. Recently, this bottleneck was solved by promoting the DIET mechanism, where electron transfer can directly proceed via bio-electrical connections or in combination with abiotic conductive materials such as biochar, activated carbon or magnetite [61]. DIET stimulates microbial communities, rapidly establishing biological electrical connections [62]. DIET was shown to reduce initial lag-phase, enhancing organic matter degradation and improving, at the same time, biogas production rate [63]. Ethanol could be used to stimulate DIET, favoring the formation of aggregates having higher conductivity and stability in response to environmental disturbances, with enrichment in *Petrimonas* and *Methanothrix* species [62]. Besides traditional conductive materials, in a recent literature study blast furnace dust was tested in UASB treatment of synthetic wastewater, highlighting an increased methane production due to an improved DIET mechanism [64].

## 4. Two-Stage UASB Anaerobic Digestion

An alternative to single stage digestion is two or multiple stage process, where in each phase the conditions are optimized for a different biomass type: typically, in the first step hydrolytic and acidogenic reactions are completed, followed by methanogenic reactions in the second stage. The microbial community operating in the AD process, in fact, can be broadly classified into acidogenic and methanogenic, whose optimal operational and environmental conditions do not coincide, mostly in terms of the pH where the maximum activity is observed [65]. Acidogenic bacteria, in fact, require a lower pH (4–6) and a shorter HRT than methanogenic bacteria [66]. 

The main advantages and drawbacks of two-stage AD are summarized in Table 2. Two-stage systems provide an optimal process stability with enhanced pollutant removal, an increased energy efficacy and an enhanced control of critical parameters, by reducing VFA and ammonia inhibition, controlling at the same time the production of harmful byproducts and providing sufficient buffering capacity [65]. On the other hand, co-digestion leads to increased capital costs and process control is not as simple as in mono-digestion operations, due to the not standardized operating conditions.

Recent studies claimed a 10–30% increase in methane yield via two-stage AD, even if this augment is typically not sufficient to sustain the costs of building a second digester in full-scale operations [67]. Through a techno-economic analysis, it was proved that two-stage AD is slightly more expensive than single-stage AD [67]. However, the economic profitability of two-stage AD should be assessed in each specific application, given the extreme variability in local market and substrate characteristics. Further work is required, in addition, to standardize two-stage AD to optimize OLR, HRT, total solids (TS)/ volatile solids (VS) balance [67]. 

Two-stage AD, with separation of acidogenesis from methanogenesis, has proved to be particularly effective in the treatment of readily biodegradable substrates such as food waste, where the second process stage can be accomplished using high-rate UASB reactor [65]. Generally, it should be reminded that acid fermentation reduces alkalinity and reactor instability is triggered when acid generation is faster than degradation through the methanogenic pathway [68]. Thus, the substrates which are known to undergo fast acidification, are the most suitable ones to apply two-stage treatment. Consistently, a two-stage system was applied to treat potato wastewater in the work in [68]. In the treatment of starch wastewater, pre-acidification was similarly shown to be beneficial for granular sludge stabilization, avoiding granules floatation and disintegration (triggered by extracellular polymeric substances, EPS), when compared to single-stage UASB system [69]. A combined two-stage CSTR-UASB digestion system achieved superior performances and an alkali-addition free cheese whey wastewater treatment, if compared to single UASB treatment [70].

Two-stage UASB treatment is beneficial also when dealing with substrates containing a fraction of readily degradable matter together with a fraction of slowly degradable compounds. In fact, a two-stage process was proposed in [71] to treat purified terephthalic acid wastewater, with acidogenic and methanogenic reactors operating at different HRTs, obtaining an enhanced pollutant abatement. More complex schemes can be adopted in particular situations, operating with different COD loading rates in the different process stages. A three-stage UASB reactor with methanogen sludge recirculation was studied in [72] for H_2_ and CH_4_ production from cassava wastewater, with a significantly higher energy yield, if compared to single and two-stage UASB reactor [72]. Coherently, H_2_ and CH_4_ were produced in two-stage UASB reactor treating cassava wastewater with added cassava residue in [73]: at a thermophilic temperature (55 °C), by applying an OLR of 10.29 kg COD/m^3^d, a favorable gas composition was highlighted both in the first (42.3% H_2_, 55% CO_2_, 2.7% CH_4_) and second (70.5% CH_4_, 28% CO_2_, 1.5% H_2_) reactor [73]. A two stage system, including a UASB reactor for H_2_ production and an IC reactor for CH_4_ production was proposed in [66] to treat medicine herbal wastewater, obtaining a H_2_ yield of 3.0 L/L d in the UASB reactor (HRT = 6 h) and a CH_4_ production of 2.54 L/L d in the IC reactor (HRT = 15 h) [66]. In optimal conditions, COD removal was 90%, with an energy conversion efficiency of 72.4% [66]. Modified UASB systems for hydrogen and VFA production are described more in depth in Section 7.

## 5. UASB Co-Digestion

Single substrate AD can lead to inhibitory phenomena, due for example to a lack in alkalinity or to an excessive ammonia concentration in the feeding stream [74]. The contemporary anaerobic treatment of multiple substrates, widely known as co-digestion process, is accepted to be beneficial for an enhanced process stability and an increased biogas generation, helping to balance macro- and micro-nutrient concentration [75]. 

Recent research focused on addition of micronutrients to enhance co-digestion performances, as well as on the contemporary treatment of substrates available in a local territory, contributing to circular economy and sustainability principles. The most recently reported UASB co-digestion results were summarized in Table 3. It can be seen that food waste and farm wastewater are commonly investigated streams [76,77,78,79], characterized by complementary properties, mostly in terms of carbon to nitrogen (C/N) ratio and nutrients, where co-digestion can lead to a significant increase in energy yield. Applied HRT in co-digestion studies is typically longer than that used for the treatment of single substrates (as reported in Table 1), while again mesophilic operations are claimed for most of the tests. 

Micro-nutrient supplementation (in form of Fe, Co, Ni, Se, Mo) was proved to be crucial to enhance methanogenic activity, stimulating methane production [76]. The addition of metals and natural elements showed to have a positive effect both on COD removal and on biogas production in UASB co-digestion of high-loaded substrates [76,77]. Carbon-rich co-substrates can be strongly beneficial, instead, when working with sulfate rich-wastewater, given the competition between sulfate-reducing bacteria (SRB) and methanogenic biomass [80], which is reduced by C addition. On the other hand, nutrient and alkalinity can be provided by animal residues when working with highly degradable and acidifying substrates [81]. An integrated solution for the treatment of different farm wastes, such as the liquid fraction of manure and cheese whey, characterized by complementary properties, was recently proposed and could be applied in areas with intense presence of cheese factories and intensive livestock farming, reducing overall environmental pollution [82]. 

From the data reported in Table 3, a great effort in finding suitable substrates for co-digestion in UASB reactor emerges, even if most of the studies are conducted at laboratory phase and need a further deepening to prove up-scale feasibility. Moreover, the analysis of available waste and wastewater fluxes in a selected area is recommended, estimating the total potential energy production and allowing continuous operations throughout the year. In fact, not all the industrial waste and wastewater streams are produced in a continuous way: as an example, ethanol industry produces sugarcane molasses and vinasses, whose co-digestion proved to be highly beneficial, due to the factory batch operations mode [83]. 

## 6. UASB Application as Anammox Process

Anammox process consists in the simultaneous abatement of ammonium and nitrite to nitrogen gas and is performed by anaerobic ammonium oxidation bacteria [84]. Anammox is being widely applied worldwide to remove high nitrogen loads, given that the total cost of partial nitrification-Anammox process is significantly lower than that of conventional nitrification-denitrification processes [84]. In fact, conventional biological nitrogen removal (BNR) processes are economically limited by the large amount of excess sludge that is inevitably produced [85]. It was recently proved that Anammox process is optimized by growing Anammox bacteria in granular form, enhancing biomass retention and shock resistance, as well as system resilience [84]. 

Landfill leachate is a complex wastewater with high levels of COD and ammonia, with a tendency to COD reduction and increase in NH_4_^+^-N over time: the treatment of mature landfill leachate is particularly cumbersome due to the low COD/N ratio [86]. In a recent literature work, a three-stage Simultaneous Ammonium oxidation Denitrifying (SAD) process was proposed to treat mature landfill leachate [85]. UASB was intended as Anammox reactor, efficiently removing ammonia and nitrite (formed in the previous process stage). An excellent TN removal (98.3%) was obtained in the integrated process, with reduction in oxygen consumption, sludge production and organic matter concentration, if compared to traditional aerobic treatment [85]. Similarly, in the work in [86] UASB reactor was used as Anammox process to treat landfill leachate after a pre-denitrification phase, fully degrading biodegradable COD in wastewater and reducing the need for oxygen supply in the aerobic process. 

It is known that the activity of Anammox bacteria strongly depends on the operating temperature. An Anammox UASB system operating at ambient temperature (between 9 °C and 28 °C) was proposed in [87] for artificial wastewater treatment without temperature control, underlining high N removal ability (90%) during Summer period, with a maximum N removal rate up to 62.5 kg N/m^3^d and an enrichment of Anammox bacteria in the UASB granular sludge [87]. An external nitrite source was used instead in an Anammox UASB reactor treating diluted chicken waste digestate, characterized by high presence of nitrogenous and organic compounds in the work in [88], obtaining a good pollutant abatement (respectively, 57% on total ammonia nitrogen and 80% on COD).

## 7. Modified UASB Systems for Bio-Hydrogen, Volatile Fatty Acids and Methane Production

The original UASB configuration was modified in a number of scientific studies to satisfy a particular purpose, such as producing hydrogen or VFA (rather than methane), or increasing reactor performances through the introduction of packing materials (Table 4). UASB reactor, in fact, can be used also for bio-hydrogen production by inhibiting methanogenic bacteria through sludge thermal pre-treatment, acid-basic procedures or headspace gas recirculation [89,90,91]. Different substrates have been tested for bio-hydrogen production, including palm oil mill effluent, winery wastewater and synthetic media [89,90,91]. Besides H_2_, waste-derived VFA, especially acetate, are valuable bio-refinery products that can be used as precursors to fuels and chemicals in different industrial sectors [92]. In particular, foul condensate from a Kraft pulp mill was shown to be adapt for VFA production in a UASB reactor, due to its high methanol, ethanol and acetone content [92].

The presence of support materials in a traditional UASB reactor for methane production triggers the formation of densely packed aggregates and a more consistent presence of large granular sludge (≥0.6 mm), with an increase in EPS production, promotion of VFA degradation and methane yield stimulation, together with stabilized performances [93,94]. Different materials were proposed for packing UASB reactor, including metals, minerals, recycled plastic material, synthetic grass and biochar [93,94,95,98,99]. Biochar, in particular, is a biomass-derived carbonaceous material, obtained from pyrolysis process, with proved capability of enhancing AD process performances, by DIET stimulation, C/N ratio optimization, micro-pollutant adsorption [74]. Biochar is able to enhance UASB performances when treating highly soluble substrates, that rapidly produce VFA: in a recent study, a biochar-amended UASB reactor treating diluted food waste paste highlighted a significantly higher COD removal than control reactor (77% versus 47%), with improved biogas yield at an OLR of 6.9–7.8 kg COD/m^3^d [99]. 

Hybrid aerobic-anaerobic reactors, coupling a UASB section and a packed bed reactor, were investigated in the work in [96] for swine wastewater treatment, with a progressive OLR increase, allowing nitrogen removal in the final aerobic phase. This solution would allow complying with current regulations for discharge to water bodies [96]. Micro-aerated UASB reactor can be a feasible solution to reduce effluent toxicity, in particular when treating complex industrial wastewater: micro-aeration allows removing the aromatic amines formed under anaerobic conditions [97]. 

## 8. UASB Treatment of Municipal Wastewater

The operating temperature is known to have a primary impact on anaerobic process kinetics (Section 2.2): low-temperature psychrophilic operations are particularly critical when treating diluted or refractory wastewater streams in a UASB reactor. In a consistent way, recently a low methanogenic and sulfidogenic activity was reported at temperatures of 17.5–25 °C in UASB treatment of sulfate-rich methanol wastewater (COD/SO_4_^2−^ ratio of 0.5), while an excellent COD removal (>90%) was obtained at 40–50 °C, with methanogens prevalence over sulfidogens [100].

In order to reduce UASB reactor heat requirement, in particular in cold climates, low-temperature digestion could be implemented by proper setting of the operating parameters, improving the overall energy balance. Considering a plant-wide perspective, in fact, in psychrophilic operations a higher amount of potential energy is available to be exploited for electricity or heat production. In recent literature studies, low-temperature (15 °C) UASB treatment of municipal sewage was proved to be technically feasible, in particular in co-digestion with rapidly degradable co-substrates (such as glucose), with moderate COD removal efficiency (23%) and triple specific methanogenic activity, if compared to sewage mono-digestion [101]. The main challenge in low-temperature UASB treatment of municipal wastewater consists in the slow hydrolysis kinetics of complex and suspended material, as well as in the slow methanogen growth [101]. 

In ambient temperature operations, a major impact is due to temperature fluctuations, that can be particularly limiting in the Winter period for biomass activity, and to reactor configuration, that has to be properly engineered to enhance applicable OLR and biogas yield [102,103]. A meaningful study [102] investigated low-temperature (10–20 °C) UASB treatment of domestic wastewater at short HRT (6 h). A stable COD removal (mean 60%) and a low effluent COD concentration (mean 90 mg/L) were claimed at temperatures ranging from 12.5 and 20 °C, with CH_4_ production evaluated as 39.7% of the influent COD, while decreased performances were reported at 10 °C [102]. In another study the feasibility of low-temperature anaerobic operations still appeared somehow challenging: in dairy wastewater treatment through UASB and EGSB reactors (OLR of 7.5–9 kg COD/m^3^d), it was shown that UASB performances were better than those of EGSB reactor [103].

Regarding low-temperature high-rate anaerobic treatment, it should be reminded, in addition, that lower operating temperatures increase methane solubility in water, leading to significant energy losses, due to the presence of dissolved methane in the effluent [104]. The use of sequential Down-flow Hanging Sponge (DHS) was recently suggested to recover a high fraction (57–88%) of dissolved CH_4_ from UASB reactor effluent, improving at the same time treated water quality, due to 90% abatement of suspended solids and COD [104]. Membranes could be used, as well, to recover methane from the treated anaerobic effluent, even if a higher fouling propensity was claimed in UASB effluent (rather than AnMBR) in the work in [105], showing that the majority of foulants were protein-like substances.

A number of full-scale UASB plants are in operation worldwide without reactor heating, to reduce energy expenses and simplify daily operations. Regarding process efficiency, a long-term analysis of seven full-scale treatment plants operating at ambient temperature in India highlighted that UASB reactor, followed by conventional aerobic processes, failed to comply with required effluent standards, while UASB reactor, followed by a DHS system, allowed to obtain high COD, BOD, total suspended solids (TSS), ammonia and phosphate abatements [106] and thus could be considered a more environmentally friendly solution. Similarly, full-scale UASB treatment at ambient temperature (mean 17 °C) was reported in the work in [107] in Bolivia, with moderate COD and TSS abatements, even if biogas valorization was not done [106]. This proves that an advancement is needed in developing countries to efficiently exploit the high-energy content of biogas. In some cases, in fact, biogas is flared, without any energy recovery, or even dispersed in the atmosphere, with negative environmental effects due to huge greenhouse gases (GHG) emissions that contribute to global temperature increase.

The study and application of decentralized wastewater treatment systems is necessary to develop sustainable water management practices in small and remote communities, not served by public sewer networks. Among the available technologies, it is widely accepted that UASB process has significant advantages over aerobic treatment, including reduced capital investment, lower energy demand, reduced sludge production and biogas exploitation [108]. Energy recovery from decentralized wastewater streams provides a significant contribution to GHG emission reduction, promoting at the same time resource recovery [109]. Source-diverted blackwater, in particular, collected from vacuum toilets, is an ideal stream for AD application, given its high organic and solid content [109]. 

Recently, it was proved that the UASB reactor could be applied as a decentralized system, co-digesting separated blackwater and kitchen waste, with an overall reduction in environmental impact (considering water, fertilizers and emissions) if compared to different alternative scenarios, including septic tanks followed by composting and septic tanks followed by landfill [110]. Similarly, UASB reactor was tested to treat blackwater (COD concentration of 1.00 g/L) in the work in [111], by applying a HRT of 2.2 d at 35 °C temperature: COD removal efficiency was above 80%, even if some methanogenesis inhibition was observed due to SRB competition [111]. High efficiency blackwater treatment was reported also in [109], where an OLR of 4.1 kg COD/m^3^d was applied (HRT of 2.6 d), obtaining a COD removal efficiency of 84% and a good methane production of 0.68 m^3^ CH_4_/m^3^d. The positive reported outcome was related to the prevalence of H_2_/CO_2_ methanogenic pathway [109]. To compare the effect of diverse influent streams, UASB was used to treat both blackwater and sanitary wastewater in the work in [112], highlighting a higher COD abatement when treating blackwater (77% versus 60%). The good COD removal was maintained despite the fluctuating OLR, coupled with a moderate *E. coli* (1.96 log) and Total Coliforms (2.13 log) removal [112]. In a coherent way, a recent notable research proved that different blackwater types (coming from conventional and vacuum toilets) enriched distinct microbial consortia in UASB treatment, probably due to the diverse sulfate and ammonia loadings [113].

Another literature work showed that UASB reactor can be seen as a second treatment stage (after septic tank) for onsite wastewater treatment in developing countries, with high TSS (83%) and COD (88%) removal efficiencies [114]. Primary septic tank presence allows to reduce the required UASB start-up time [114]. A more complex scheme for raw municipal wastewater treatment, including a UASB reactor followed by a settler (having HRT of 3 h, where sludge was concentrated) and a digester, was proposed in the work in [115] to improve global energy efficiency of the system. The integrated process had total HRT of 6 h, achieving a mean COD removal of 49.2% [115]. Compared to single UASB reactor, the combined UASB-settler-digester system had similar COD abatement and methane production applying a reduced sludge recirculation rate, leading to 50% energy saving [115]. 

More advanced technological approaches, focused on resource recovery (beside energy recovery from biogas) involve the addition of calcium in a modified UASB treatment (with internal gas lift, GL) of vacuum collected blackwater, to increase total phosphorous (TP) retention [116]. An excellent COD (92%) and P (90%) removal was claimed, together with recovery, at the same time, of CaP granules (>0.4 mm diameter), containing an average of 7.8% P [116]. A potential 57% P recovery in source-separated sanitation was calculated in the combined UASB-GL process [116]. It should be reminded that P recovery is highly recommended to be implemented in wastewater treatment in the future, considering that P is an essential element for living organisms and that mineral-derived P is predicted to be depleted within the next 100 years [117].

It could be concluded that a number of research studies proved the feasibility of municipal wastewater treatment in UASB reactor, that is particularly efficient if innovative collecting techniques (such as vacuum sanitation) are applied, leading to an increased OLR and a consequently higher biogas generation, together with a reduced freshwater usage. Material recovery, particularly important in the case of phosphorous, can be coupled with energy recovery, enhancing the sustainability of wastewater treatment. Finally, in order to achieve 100% of clean and renewable energy, UASB process in decentralized areas could be coupled with solar thermal energy, in particular in hot climate areas. Through a modeling approach, it was recently demonstrated that a 100% energy saving could be obtained with an integrated UASB-solar system in Morocco for 10 months per year, while 70% energy saving could be reached in the residual two cold months per year [118]. In addition, it was proved that the operating temperature in the digester never dropped out of the mesophilic range, even in the absence of solar production (due to a poor irradiation) [118].

## 9. UASB Pre- and Post-Treatment

As previously mentioned, a number of different pre-treatments can be applied before AD to augment methane yield, depending on substrate characteristics and physicochemical composition: the applicable pre-treatments have been widely investigated in recent literature studies, both on traditional AD systems and advanced UASB reactors. The possible techniques to increase substrate biodegradability include physical (size reduction), chemical (acid, alkaline, organic solvent), thermophysical (hot water, steam explosion, ultrasound, microwave), thermochemical (wet oxidation, supercritical CO_2_) and biological (microbial, enzymatic) methods [119]. 

Sewage contains 30–70% of particulate COD, degrading at a slower rate than soluble organic fraction: a pre-hydrolysis phase ensures better UASB performances in sewage treatment at an increased OLR, in particular if using two-stage systems [120]. Lignocellulosic streams can be efficiently pre-treated with acid and enzymatic methods, even if industrial scale application of these methods is still limited [4]. Temperature pre-treatment and temperature-phased advanced AD were lately studied to facilitate veterinary antibiotic degradation [121], that is characterized by a significant refractory fraction. In another work, chemical pre-treatment of onion waste was proposed in a packed bed reactor coupled with a UASB reactor to reduce VFA accumulation and improve biogas yield [122]. Sulfuric acid pre-treatment, in particular, allowed to decrease the required start-up time, with methane yield up to 0.41 m^3^ CH_4_/kg VS_removed_ [122]. Mechanical solid-liquid separation of organic fraction of municipal solid waste (OFMSW), instead, allows to obtain a highly-degradable liquid fraction, called press water or leachate, able to produce consistent methane yields in high-rate systems, such as UASB reactor [16]. Ultrasound (US) and *Escherichia Coli*/*Aspergillus Niger* biodegradation pre-treatments were demonstrated to be efficient in reducing long-chain fatty acids (LCFA) concentration in UASB treatment of crude glycerol, reducing methanogenic bacteria inhibition, with methane yield increase up to 29% (with US pre-treatment) and 77% (with *A. Niger* pre-treatment) [123]. Furthermore, recently an emulsification pre-treatment was shown to be efficient for high-rate anaerobic treatment of an oleic acid based effluent [124].

Enzymatic pre-treatment is an environmental-friendly technique, if compared to chemical methods, and was proposed in the work in [125] to increase biogas yield from palm oil mill effluent (POME). A complex treatment scheme for oilfield wastewater treatment was instead proposed in the work in [126], including dissolved air floatation, yeast bioreactor, UASB reactor and biological aerated filter, showing excellent COD, suspended solids and oil removal. UASB reactor, in particular, enhanced effluent biodegradability for the final aerobic phase [126]. The most recent pre-treatment methods are basically aimed at increasing substrate biodegradability, in the case of lignocellulosic and refractory streams, or at reducing acid accumulation in the system, that leads to an imbalance in the anaerobic trophic chain, with acidogenesis prevalence over methanogenesis. Besides evaluating the effectiveness of the investigated pre-treatment method, the most important aspect to be assessed when upscaling a pre-treatment is to evaluate its economic and energy sustainability: the obtained augment in methane yield should be sufficient to cover the extra costs for mechanical equipment installation and energy (or chemical) consumption of the pre-treatment phase. Moreover, the additional environmental impact has to be studied, due to the usage of chemical compounds and to substrate degradation.

On the other hand, a post-treatment is typically required on effluents from UASB reactor to comply with the required legislative standards, especially regarding nutrients, pathogens and solids [12]. Recently, high-rate algal ponds (HRAP) were proposed as an innovative post-treatment for a UASB effluent operating at environmental temperature [15]. UASB co-treated raw sewage and harvested microbial biomass from HRAP reactor: overall COD and NH_4_-N removals of 65% and 61% were claimed, respectively, in the co-digestion system, with 25% increase in CH_4_ yield (from 156 to 211 NL CH_4_/kg VS) if compared to UASB treatment of sewage alone [15]. UASB reactor was followed by an aerobic process in the work reported in [127] in recalcitrant azo-dye treatment, showing high COD (92.4%) and color (98.4%) removal at HRT of 6 h, corresponding to an OLR of 12.97 g COD/L·d, with maximum CH_4_ yield of 13.3 mmol CH_4_/g COD d [127]. 

Beside the traditional activated sludge process, an efficient post-treatment of anaerobic UASB effluents could be achieved by applying sequencing batch reactors (SBR) with granular aerobic sludge, even if particular attention has to be put in reactor start-up [128], similarly to what happens in anaerobic granular systems. A pilot system consisting of a UASB reactor and a successive microalgae post-treatment (*Chlorella sorokiniana* cultivated in three flat photobioreactors) was tested in the work reported in [129] to treat a mixture of municipal and piggery wastewater, obtaining a high organic removal efficiency, despite fluctuations in the influent characteristics. Microalgae abated dissolved inorganic carbon (46–56% removal), orthophosphate (40–60% abatement) and ammonia (100% efficiency) [129]. A simple surface aerator post-treatment was finally proposed in the work in [130] as an energy-saving technique after UASB treatment of municipal wastewater coming from an Indian WWTP. It could be concluded that UASB can be coupled with extremely different post-treatments, from conventional biological processes and simple aerators to advanced granular systems, depending on the required effluent quality; microalgae, in particular, have a high integration potential in WWTPs, due to the possibility to use the excess biomass for biogas production, leading to a circular cascade.

## 10. UASB Reactor and Wastewater Toxicity

Sewage sludge production is increasing worldwide due to the growing population and an improved wastewater treatment [131]. The applied techniques for sludge treatment substantially include agricultural application and thermochemical methods [131]. The environmental impact of water treatment residues should be thoroughly assessed through toxicity evaluation to allow a safe sludge application to the soil. Eco-toxicity of treated wastewater and sludge is being given attention: sewage sludge is reported to contain at hazardous concentrations heavy metals, pharmaceuticals and personal care products [131]. As previously stated, UASB treatment abates nutrients, biological agents and trace metals in a limited way [132], with accumulation in the residual solid fraction. Heavy metal pollution poses a serious threat for human and environmental health, if not effectively removed from wastewater [5]. Coherently, a recent study highlighted that UASB treatment, followed by an aerobic bioreactor, is not adequately efficient for acute toxicity abatement in municipal wastewater treatment, with final toxicity values ranging from non-toxic to moderately toxic [133]. The applied wastewater treatment processes should consider not only metal removal efficiency, but also the potential to promote the most preferable phase distributions [132]. Metals in UASB sludge demonstrated a high binding potential with coexisting anions, including carbonates, hydroxyls and bicarbonates, with possible negative environmental relapses [132]. 

The evaluation of trace metal composition is particularly important for sludge agricultural reutilization, considering that nowadays agricultural application is still a commonly applied technique for sludge coming from municipal wastewater treatment [5]. To this purpose, six different sewage sludges from UASB reactor were characterized in the work in [134], considering ten metals (Ni, Mn, Se, Co, Fe, Zn, K, Cu, Pb, Cr). Se, Zn, Ni and Fe were found at high percentage in the sulfide-organic matter fraction, while Co and K were mostly present in the exchangeable and carbonate fractions and Pb appeared in the residual fraction [134]. In a consistent way, a high capability of UASB reactor to remove selenate in presence of cadmium and zinc was reported in the work in [135] under psychrophilic and mesophilic conditions, with excellent removal efficiency, coupled with very high Cd(II) and Zn(II) abatements (particularly in the mesophilic range). 

Another critical aspect to be considered in wastewater treatment, as previously mentioned, is the presence of pharmaceutical residuals, which pose severe risks to the receiving environment, if not removed from wastewater. Antibiotics, in particular, have been detected in water, soil, manure and sludge, due to the low removal efficiency commonly observed in conventional pharmaceutical WWTPs [136]. The application of advanced oxidation processes (AOPs) is being currently studied, considering that the highly reactive species produced in AOPs can destroy antibiotics and deactivate microbes, together with refractory compounds [136,137]. In this framework, a complex treatment scheme, including a UASB reactor, an anoxic-oxic tank and a final AOP (UV, ozonation, Fenton, Fenton/UV) was proposed in the work in [136] to simultaneously remove 18 antibiotics and 10 antibiotic resistant genes. Interestingly, UASB reactor gave the most significant contribution (85.8%) in pharmaceutical abatement, with mechanisms including both degradation and sorption to sludge [136]. A similar complex treatment scheme, with a UASB treatment followed by an aerobic sequencing batch reactor (SBR) and a final Fenton-like oxidation process, was proposed in [138] to abate antibiotics (highly concentrated in swine wastewater). UASB was efficient in abating organic contaminants (COD removal of 75%) while SBR and Fenton-like processes removed antibiotics (efficiencies, respectively, >95% and 74%), allowing for reutilization of treated wastewater as farm fertilizer [138]. It could be concluded that, while UASB treatment shows a good abatement even of hazardous and refractory emerging contaminants, an accumulation in the sludge fraction of these compounds is observed: a thorough characterization of the material is consequently required before land application, to avoid negative environmental effects.

## 11. Critical Aspects and Future Perspectives

Biogas from AD is a relevant renewable energy source that plays a significant role in environmental pollution mitigation and local electricity production [139]. While most of the available literature studies focus on UASB application to different substrates at laboratory or pilot scale, by analyzing process efficiency and biogas production, a broader focus is needed to evaluate techno-economic scale-up feasibility, together with the possibility to integrate biogas with other renewable energies, such as photovoltaic or wind energy. A more sustainable approach in the water-energy nexus is desirable, in particular in developing regions such as Latin America [140], with efficient biogas exploitation. 

Methodologies based on life cycle assessment (LCA) and criteria indicators in sustainability studies allow to evaluate and compare different scenarios to optimize the energy and environmental aspects in biogas exploitation [140] and could be integrated with a detailed process modeling. The application of existing powerful modeling devices, in fact, helps to increase plant efficiency, fixing the most critical energy wastages. Nowadays, there exist hundreds of full-scale UASB reactors in various areas of the tropical world, in particular in India and Latin America (especially Brazil) [141]. However, operating problems, including scum accumulation in the settler compartment and in the gas-liquid-solid separator, as well as odor nuisance, often pose a significant threat to maximize reactor performances [141]. Harmful H_2_S emissions due to the reduction of sulfate contained in municipal wastewater should be measured and abated [142]. An in-depth monitoring campaign was carried in full-scale UASB plants in Brazil in the work in [141] to assess these aspects, proposing a basic UASB operation optimization.

A simplified wastewater treatment scheme, including a UASB reactor followed by constructed wetlands, could be a feasible solution for developing countries, due to its simplicity and ease of operations [143]. To this purpose, a LCA approach was recently proposed to evaluate a Brazilian full-scale WWTP, consisting in a UASB reactor and a successive wetland treatment, including also reactor construction costs: a negative impact of air emissions from UASB reactor on global warming was again highlighted [143]. Consequently, a careful monitoring and a proper treatment unit of odor emissions from anaerobic reactors is recommended to avoid negative consequences also from a public perception perspective [16]. Different indicators (environmental, social and economic parameters) were proposed to assess the technical and economic suitability of a UASB reactor integrated with a down-flow hanging sponge (DHS) for sewage treatment, comparing this solution to other full-scale wastewater treatment schemes commonly found in India [144]. It was proved that UASB-DHS process had the highest value of the global sustainability indicator. In addition, a trade-off between an environmental-friendly wastewater treatment and the socio-economic aspects was highlighted [144].

Energy analysis is particularly important to assess the full-scale anaerobic treatment feasibility and the capability of the produced biogas to fulfil a significant share of plant energy requirement. The energy potential of sludge and biogas in a Brazilian WWTP (70,000 population equivalent) was studied in the work in [145], claiming that electricity produced from biogas could supply up to 57.6% of total WWTP energy demand [145]. Multivariate analysis in wastewater treatment can be a meaningful tool to reduce operating costs [146]. As an example, the application of Principal Component Analysis (PCA) in swine wastewater treatment (consisting in a UASB reactor followed by aerobic filters and wetland) allowed to increase COD removal efficiency from 45% to 67% [146].

The most important advantages and critical aspects for further UASB application, in light of the results of recent literature studies, were briefly summarized in Table 5. A notable interest was found in UASB reactor application to a pool of different high-loaded substrates, focusing on the treatment of recalcitrant wastewater (by selection of modified UASB systems or complex process schemes) and on low-temperature decentralized UASB treatment, to allow a better exploitation of this technology in remote and rural areas. Advanced high-rate systems, such as IC and ICX reactors, were specifically targeted to enhance treatable organic loads and to optimize the separation between sludge, effluent and biogas. Beside methane, also hydrogen and VFA can be produced in UASB systems, leading to biorefineries development. At the same time, an efficient and compact nitrogen removal can be performed through Anammox process. A significant number of studies related treatment efficiency and biogas production to microbial community composition, giving profound insights in the process understanding. The integration of biogas with other renewable energy sources (such as photovoltaic and wind energy), despite not being often considered, could lead to local 100% renewable energy communities. Biogas and solar energy integration is highly recommended in the future to allow a significant reduction in GHG emissions, mitigating climate change and following the proposed sustainable development goals (SDG). However, attention has to be put in the residual toxicity of sludge and treated wastewater, not to compromise the receiving environment, focusing both on traditional (heavy metals, microbial indicators) and emerging (pharmaceuticals, microplastic) contaminants.

## 12. Conclusions

High-rate up-flow anaerobic sludge blanket (UASB) reactor is being increasingly applied worldwide to produce renewable energy in biogas form from a variety of wastewater streams, including both industrial and municipal substrates. In this review, the most recent advances regarding UASB application were critically presented and discussed, focusing on the most important engineering aspects for further full-scale development. It was demonstrated that UASB reactor is able to effectively treat not only highly biodegradable substrates, but also recalcitrant or diluted fractions, by selection of modified UASB systems, multi-stage anaerobic process, proper substrate pre-treatment. Anaerobic microbial community composition has been intensively studied, being sensitive to operating conditions and treated substrate. In addition, an improvement in understanding the complex hydrodynamic UASB behavior has been recently accomplished for powerful modeling purposes. Co-digestion between different substrates, available in the same area, allows increasing the obtainable methane yield, stabilizing reactor operations, due to an improved macro- and micro-nutrient balance. Furthermore, UASB has shown to be effective in nitrogen removal, when employed as Anammox treatment. A particular deepening was made on low-temperature decentralized UASB treatment of municipal wastewater, which is able to locally integrate electricity with end users, creating efficient and sustainable renewable energy clusters. As for toxicity aspects, UASB was shown to be effective in reducing heavy metal concentration and in some cases antibiotics, even if its disinfection efficiency is known to be limited. Most of the recent studies report laboratory or pilot-scale trials and consequently a further effort is required to prove full-scale energy and economic sustainability of the proposed solutions. Finally, it was concluded that the integration of biogas with other renewable energies, such as wind and photovoltaic energy, needs to be encouraged, with an integration between process and energy models, in order to create 100% renewable communities and reduce greenhouse gases emissions.

## Figures and Tables

**Table 1:** Reported recent literature studies on UASB treatment of high-loaded substrates.

Substrate	Temperature (°C)	Influent Chemical Oxygen Demand (g/L)	Chemical Oxygen Demand Removal (%)	Hydraulic Retention Time (h)	Organic Loading Rate (g COD/L·d)	Methane Yield	Reference
Glutamate-rich wastewater	35	2.0	95.5–96.5	4.5–6	8.26–10.82	0.31 ^1^	[25]
Monosodium glutamate	35	7.9	97	24	8	2.3 ^2^	[26]
Sugarcane bagasse hydrolysate	20–30	1.82	86	18.4	2.4	0.27 ^1^	[27]
Recycled paper mill wastewater	37	5.7	80.6	15.14	5.18	0.89 ^2^	[28]
Vinasse	Ambient	120.2	91–93	40	72.1	0.46–0.53 ^2^	[18]
Guar	37	1.1	79–84	10	2.78	0.15–0.16 ^3^	[29]
Synthetic fiber wastewater	13.9–32.1	1.7–30.7	75.8	24	1.3–21.5	0.4–2 ^2^	[30]
Pistachio wastewater	35	49.8	89.8	5.4 d	4.56	0.33 ^1^	[31]
Perchlorate	30	-	84.7	2.2	9.96	-	[32]
Synthetic slaughterhouse wastewater	37	1.7	70	10	3.94–8.15	0.35 ^2^	[33]
Chocolate wastewater	15–30	6.2	39–94	6	2–6	0.3–1.9 ^4^	[34]
Pig slurry	36	21.5	-	1.5 d	14.3–16.4	0.25 ^1^	[35]
Leachate from waste incineration	35	36.8	97.5–99.5	1.3–3 d	1.86–7.43	-	[36]

^1^ L CH_4_/g COD, ^2^ L CH_4_/L·d, ^3^ L CH_4_/g COD d, ^4^ L biogas/L·d.

**Table 2:** Main advantages and drawbacks of two-stage UASB treatment.

Advantages	Drawbacks
Improved process stability	Increased capital costs
Increased pollutant abatement	Not standardized operating conditions (Hydraulic Retention Time, Organic Loading Rate, total solids (TS)/ volatile solids (VS)
Augmented methane yield	
	Need for a second digester
Optimal operating conditions for diverse microorganism consortia	Biogas surplus typically not sufficient to cover expenses
Better process control	Need for an extra process control
Reduced fatty acid and ammonia inhibition	Economic sustainability not always favorable
Augmented buffering capacity	
Improved control of byproducts	
Enhanced sludge stabilization	
Reduced biomass floatation and disintegration	
Effective in readily biodegradable substrate treatment	

**Table 3:** Recently reported UASB co-digestion results.

Substrate	Co-Substrate	Adjuvant	Temperature (°C)	Hydraulic Retention Time (d)	Organic Loading Rate (kg COD/m^3^d)	Chemical Oxygen Demand Removal (%)	Yield	Reference
Food waste	Domestic wastewater	Cu^2+^	35	10	3.8	>90	0.3 ^1^	[76]
Food waste	Liquid slaughterhouse waste	Clinoptiolite	40	28	-	-	-	[77]
Cheese whey	Manure liquid fraction	None	35	2.2	19.4	95	6.4 ^2^	[82]
Distilled gin	Swine wastewater	None	36	3.3	28.5	97	8.4 ^2^	[81]
Sewage sludge-cow manure	Kitchen waste, yard waste, floral waste, dairy wastewater	None	36	1	-	78–86	4.5 ^2^	[78]
Acid mine drainage	Cheese whey	None	30	1	-	68–84	-	[80]
Source-diverted blackwater	Food waste	None	35	2.6	10.0	82–84	2.42 ^3^	[79]

^1^ L CH_4_/g COD_removed_, ^2^ m^3^ CH_4_/m^3^d, ^3^ L/L·d.

**Table 4:** Recently reported UASB modified systems.

Substrate	Reactor	Temperature (°C)	Product	Hydraulic Retention Time (d)	Organic Loading Rate (kg COD/m^3^d)	Yield	Reference
Palm oil mill effluent	UASB	55	H_2_	0.25	-	11.75 ^1^	[89]
Winery wastewater	UASB	37	H_2_	0.23	-	62 ^2^	[91]
Synthetic media	UASB	-	H_2_	0.25	-	4.34 ^1^	[90]
Foul condensate from Kraft mill	UASB	22–55	VFA	3.13	8.6	52–70 ^3^	[92]
Nitrobenzene	UASB with nanoscale zero-valent iron addition	35	CH_4_	1	0.2 ^4^	-	[93]
Petroleum wastewater	UASB with diatomite and maifanite addition	36	CH_4_	10–20	11	1.61–2.2 ^5^	[94]
Sewage water	Packed UASB reactor	Ambient	CH_4_	0.21–0.25	1.8	-	[95]
Swine wastewater	UASB+ aerobic packed bed reactor	37	CH_4_	0.79	3.26–10.14	0.26–0.81 ^6^	[96]
Textile wastewater	Micro-aerated UASB	25	CH_4_	-	1.27–1.5	-	[97]
Cattle slaughterhouse wastewater	UASB with synthetic grass packing	35	CH_4_	1	10	2.0 ^5^	[98]
Diluted food waste paste	UASB with biochar addition	30	CH_4_	1	6.9–7.8	0.86 ^7^	[99]

^1^ L H_2_/L_effluent_ d, ^2^ mL H_2_/L h, ^3^ % of utilized carbon, ^4^ kg NB/m^3^d, ^5^ m^3^/m^3^d, ^6^ kg COD-CH_4_/kg VSS d, ^7^ L biogas/g COD_removed_ d.

**Table 5:** Advantages and critical aspects of UASB application, according to recent literature studies.

Advantages	Critical Aspects
Possibility to get clean energy in decentralized areas	Not efficient disinfection and nutrient removal
Proved efficiency on high-loaded biodegradable streams	Residual effluent toxicity in the sludge to be carefully evaluated
	Low-temperature operations not efficient on diluted streams
Modified UASB systems allow to efficiently treat refractory streams	Limited integration with other renewable energy sources
Co-digestion of complementary substrates in the same territory increases plant sustainability	Need for a post-treatment to abate pollutants under required law limits
Possibility to produce hydrogen and volatile fatty acids	Limited biogas valorization in developing countries
Possibility to abate N through granular Anammox process	UASB start-up phase is particularly critical
Significant reduction of excess sludge in comparison with traditional flocculent processes	Pre-treatments to increase biogas yield are not very applied at full-scale
Improved microbial understanding and use of modeling tools helps to optimize performances	Need for an efficient odor abatement

## References

[1] Rao P.V., Baral S.S., Dey R., Mutnuri S. (2010). Biogas generation potential by anaerobic digestion for sustainable energy development in India. Renew. Sustain. Energy Rev..

[2] Rasapoor M., Young B., Brar R., Sarmah A., Zhuang W.-Q., Baroutian S. (2020). Recognizing the challenges of anaerobic digestion: Critical steps toward improving biogas generation. Fuel.

[3] Kumar A., Samadder S.R. (2020). Performance evaluation of anaerobic digestion technology for energy recovery from organic fraction of municipal solid waste: A review. Energy.

[4] Gunes B., Stokes J., Davis P., Connolly C., Lawler J. (2019). Pre-treatments to enhance biogas yield and quality from anaerobic digestion of whiskey distillery and brewery wastes: A review. Renew. Sustain. Energy Rev..

[5] Misson G., Mainardis M., Incerti G., Goi D., Peressotti A. (2020). Preliminary evaluation of potential methane production from anaerobic digestion of beach-cast seagrass wrack: The case study of high-adriatic coast. J. Clean. Prod..

[6] Zhong Y., Chen R., Rojas-Sossa J.-P., Isaguirre C., Mashburn A., Marsh T., Liu Y., Liao W. (2020). Anaerobic co-digestion of energy crop and agricultural wastes to prepare uniform-format cellulosic feedstock for biorefining. Renew. Energy.

[7] Zhang L., Loh K.-C., Zhang J. (2018). Food waste enhanced anaerobic digestion of biologically pretreated yard waste: Analysis of cellulose crystallinity and microbial communities. Waste Manag..

[8] Mainardis M., Flaibani S., Trigatti M., Goi D. (2019). Techno-economic feasibility of anaerobic digestion of cheese whey in small Italian dairies and effect of ultrasound pre-treatment on methane yield. J. Environ. Manag..

[9] Bórawski P., Bełdycka-Bórawska A., Szymańska E.J., Jankowski K.J., Dubis B., Dunn J.W. (2019). Development of renewable energy sources market and biofuels in the European Union. J. Clean. Prod..

[10] Chong S., Sen T.K., Kayaalp A., Ang H.M. (2012). The performance enhancements of Upflow Anaerobic Sludge Blanket (UASB) reactors for domestic sludge treatment—A state-of-the-art review. Water Res..

[11] Latif M.A., Ghufran R., Wahid Z.A., Ahmad A. (2011). Integrated application of upflow anaerobic sludge blanket reactor for the treatment of wastewaters. Water Res..

[12] Lim S.J., Kim T.-H. (2014). Applicability and trends of anaerobic granular sludge treatment processes. Biomass Bioenergy.

[13] Tauseef S.M., Abbasi T., Abbasi S.A. (2013). Energy recovery from wastewaters with high-rate anaerobic digesters. Renew. Sustain. Energy Rev..

[14] Abbasi T., Abbasi S.A. (2012). Formation and impact of granules in fostering clean energy production and wastewater treatment in Upflow Anaerobic Sludge Blanket (UASB) reactors. Renew. Sustain. Energy Rev..

[15] Vassalle L., Díez-Montero R., Machado A.T.R., Moreira C., Ferrer I., Mota C.R., Passos F. (2020). Upflow anaerobic sludge blanket in microalgae-based sewage treatment: Co-digestion for improving biogas production. Bioresour. Technol..

[16] Mainardis M., Goi D. (2019). Pilot-UASB reactor tests for anaerobic valorisation of high-loaded liquid substrates in Friulian mountain area. J. Environ. Chem. Eng..

[17] Enitan A.M., Kumari S., Odiyo J.O., Bux F., Swalaha F.M. (2018). Principal component analysis and characterization of methane community in a full-scale bioenergy producing UASB reactor treating brewery wastewater. Phys. Chem. Earth Parts A B C.

[18] Cruz-Salomón A., Meza-Gordillo R., Rosales-Quintero A., Ventura-Canseco C., Lagunas-Rivera S., Carrasco-Cervantes J. (2017). Biogas production from a native beverage vinasse using a modified UASB bioreactor. Fuel.

[19] Mainardis M., Cabbai V., Zannier G., Visintini D., Goi D. (2018). Characterization and BMP tests of liquid substrates for high-rate anaerobic digestion. Chem. Biochem. Eng. Q..

[20] Prateep Na Talang R., Sirivithayapakorn S. (2020). Environmental impacts and economic benefits of different wastewater management schemes for molasses-based ethanol production: A case study of Thailand. J. Clean. Prod..

[21] Kong Z., Li L., Xue Y., Yang M., Li Y.-Y. (2019). Challenges and prospects for the anaerobic treatment of chemical-industrial organic wastewater: A review. J. Clean. Prod..

[22] Liang J., Wang Q., Yoza B.A., Li Q.X., Chen C., Ming J., Yu J., Li J., Ke M. (2020). Rapid granulation using calcium sulfate and polymers for refractory wastewater treatment in up-flow anaerobic sludge blanket reactor. Bioresour. Technol..

[23] Li L., Kong Z., Xue Y., Wang T., Kato H., Li Y.-Y. (2020). A comparative long-term operation using Up-flow Anaerobic Sludge Blanket (UASB) and Anaerobic Membrane Bioreactor (AnMBR) for the upgrading of anaerobic treatment of N, N-Dimethylformamide-containing wastewater. Sci. Total Environ..

[24] Geißler A., Schwan B., Dornack C. (2019). Developing a high-performance methane stage for biomass with high nitrogen loads. Renew. Energy.

[25] Chen H., Wei Y., Xie C., Wang H., Chang S., Xiong Y., Du C., Xiao B., Yu G. (2020). Anaerobic treatment of glutamate-rich wastewater in a continuous UASB reactor: Effect of hydraulic retention time and methanogenic degradation pathway. Chemosphere.

[26] Chen H., Wei Y., Liang P., Wang C., Hu Y., Xie M., Wang Y., Xiao B., Du C., Tian H. (2020). Performance and microbial community variations of a Upflow Anaerobic Sludge Blanket (UASB) reactor for treating monosodium glutamate wastewater: Effects of organic loading rate. J. Environ. Manag..

[27] Ribeiro F.R., Passos F., Gurgel L.V.A., Baêta B.E.L., de Aquino S.F. (2017). Anaerobic digestion of hemicellulose hydrolysate produced after hydrothermal pretreatment of sugarcane bagasse in UASB reactor. Sci. Total Environ..

[28] Bakraoui M., Karouach F., Ouhammou B., Aggour M., Essamri A., El Bari H. (2020). Biogas production from recycled paper mill wastewater by UASB digester: Optimal and mesophilic conditions. Biotechnol. Rep..

[29] Liang J., Wang Q., Yoza B.A., Li Q.X., Ke M., Chen C. (2019). Degradation of guar in an up-flow anaerobic sludge blanket reactor: Impacts of salinity on performance robustness, granulation and microbial community. Chemosphere.

[30] Ni C.-H., Chang C.-Y., Lin Y.-C., Lin J.C.-T. (2019). Simultaneous biodegradation of Tetrahydrofuran, 3-Buten-1-Ol and 1,4-Butanediol in real wastewater by a pilot high-rate UASB reactor. Int. Biodeterior. Biodegrad..

[31] Gür E., Demirer G.N. (2019). Anaerobic digestability and biogas production capacity of pistachio processing wastewater in UASB reactors. J. Environ. Eng..

[32] Han Y., Guo J., Zhang Y., Lian J., Guo Y., Song Y., Wang S., Yang Q. (2018). Anaerobic granule sludge formation and perchlorate reduction in an Upflow Anaerobic Sludge Blanket (UASB) reactor. Bioresour. Technol. Rep..

[33] Vidal J., Carvajal A., Huiliñir C., Salazar R. (2019). Slaughterhouse wastewater treatment by a combined anaerobic digestion/solar photoelectro-fenton process performed in semicontinuous operation. Chem. Eng. J..

[34] Esparza-Soto M., Jacobo-López A., Lucero-Chávez M., Fall C. (2019). Anaerobic treatment of chocolate-processing industry wastewater at different organic loading rates and temperatures. Water Sci. Technol..

[35] Rico C., Montes J.A., Rico J.L. (2017). Evaluation of different types of anaerobic seed sludge for the high rate anaerobic digestion of pig slurry in UASB reactors. Bioresour. Technol..

[36] Li J., He C., Tian T., Liu Z., Gu Z., Zhang G., Wang W. (2020). UASB-modified bardenpho process for enhancing bio-treatment efficiency of leachate from a municipal solid waste incineration plant. Waste Manag..

[37] Wang W., Wu B., Pan S., Yang K., Hu Z., Yuan S. (2017). Performance robustness of the UASB reactors treating saline phenolic wastewater and analysis of microbial community structure. J. Hazard. Mater..

[38] Nakasaki K., Nguyen K.K., Ballesteros F.C., Maekawa T., Koyama M. (2020). Characterizing the microbial community involved in anaerobic digestion of lipid-rich wastewater to produce methane gas. Anaerobe.

[39] Aziz A., Basheer F., Sengar A., Irfanullah, Khan S.U., Farooqi I.H. (2019). Biological wastewater treatment (anaerobic-aerobic) technologies for safe discharge of treated slaughterhouse and meat processing wastewater. Sci. Total Environ..

[40] Mannacharaju M., Kannan Villalan A., Shenbagam B., Karmegam P.M., Natarajan P., Somasundaram S., Arumugam G., Ganesan S. (2020). Towards sustainable system configuration for the treatment of fish processing wastewater using bioreactors. Environ. Sci. Pollut. Res. Int..

[41] Barrera E.L., Spanjers H., Romero O., Rosa E., Dewulf J. (2020). A successful strategy for start-up of a laboratory-scale UASB reactor treating sulfate-rich sugar cane vinasse. J. Chem. Technol. Biotechnol..

[42] Lu X., Zhen G., Ni J., Hojo T., Kubota K., Li Y.-Y. (2016). Effect of influent COD/SO42—Ratios on biodegradation behaviors of starch wastewater in an Upflow Anaerobic Sludge Blanket (UASB) reactor. Bioresour. Technol..

[43] Mora M., Lafuente J., Gabriel D. (2020). Influence of crude glycerol load and pH shocks on the granulation and microbial diversity of a sulfidogenic upflow anaerobic sludge blanket reactor. Process Saf. Environ. Prot..

[44] Li H., Han K., Li Z., Zhang J., Li H., Huang Y., Shen L., Li Q., Wang Y. (2018). Performance, granule conductivity and microbial community analysis of Upflow Anaerobic Sludge Blanket (UASB) reactors from mesophilic to thermophilic operation. Biochem. Eng. J..

[45] Tassew F.A., Bergland W.H., Dinamarca C., Bakke R. (2020). Influences of temperature and substrate particle content on granular sludge bed anaerobic digestion. Appl. Sci..

[46] Zhang L., Ban Q., Li J., Wan C. (2019). Functional bacterial and archaeal dynamics dictated by pH stress during sugar refinery wastewater in a UASB. Bioresour. Technol..

[47] Vasconcelos E.A.F., Santaella S.T., Viana M.B., dos Santos A.B., Pinheiro G.C., Leitão R.C. (2019). Composition and ecology of bacterial and archaeal communities in anaerobic reactor fed with residual glycerol. Anaerobe.

[48] Debik E., Coskun T. (2009). Use of the Static Granular Bed Reactor (SGBR) with anaerobic sludge to treat poultry slaughterhouse wastewater and kinetic modeling. Bioresour. Technol..

[49] Wang T., Huang Z., Ruan W., Zhao M., Shao Y., Miao H. (2018). Insights into sludge granulation during anaerobic treatment of high-strength leachate via a full-scale IC reactor with external circulation system. J. Environ. Sci..

[50] Diamantis V., Aivasidis A. (2018). Performance of an ECSB reactor for high-rate anaerobic treatment of cheese industry wastewater: Effect of pre-acidification on process efficiency and calcium precipitation. Water Sci. Technol..

[51] Hendrickx T.L.G., Pessotto B., Prins R., Habets L., Vogelaar J. (2019). Biopaq®ICX: The next generation high rate anaerobic reactor proves itself at full scale. Water Pract. Technol..

[52] Parker W.J. (2005). Application of the ADM1 model to advanced anaerobic digestion. Bioresour. Technol..

[53] Lorenzo-Llanes J., Pagés-Díaz J., Kalogirou E., Contino F. (2019). Development and application in aspen plus of a process simulation model for the anaerobic digestion of vinasses in UASB reactors: Hydrodynamics and biochemical reactions. J. Environ. Chem. Eng..

[54] Chen Y., He J., Mu Y., Huo Y.-C., Zhang Z., Kotsopoulos T.A., Zeng R.J. (2015). Mathematical modeling of Upflow Anaerobic Sludge Blanket (UASB) reactors: Simultaneous accounting for hydrodynamics and bio-dynamics. Chem. Eng. Sci..

[55] Brito M.G.S.L., Nunes F.C.B., Magalhães H.L.F., Lima W.M.P.B., Moura F.L.C., Farias Neto S.R., Lima A.G.B. (2020). Hydrodynamics of Uasb reactor treating domestic wastewater: A three-dimensional numerical study. Water.

[56] Tsui T.-H., Ekama G.A., Chen G.-H. (2018). Quantitative characterization and analysis of granule transformations: Role of intermittent gas sparging in a super high-rate anaerobic system. Water Res..

[57] Owusu-Agyeman I., Eyice Ö., Cetecioglu Z., Plaza E. (2019). The study of structure of anaerobic granules and methane producing pathways of pilot-scale UASB reactors treating municipal wastewater under sub-mesophilic conditions. Bioresour. Technol..

[58] Callejas C., Fernández A., Passeggi M., Wenzel J., Bovio P., Borzacconi L., Etchebehere C. (2019). Microbiota adaptation after an alkaline pH perturbation in a full-scale UASB anaerobic reactor treating dairy wastewater. Bioprocess Biosyst. Eng..

[59] Show K.-Y., Yan Y., Yao H., Guo H., Li T., Show D.-Y., Chang J.-S., Lee D.-J. (2020). Anaerobic granulation: A review of granulation hypotheses, bioreactor designs and emerging green applications. Bioresour. Technol..

[60] Na J.-G., Lee M.-K., Yun Y.-M., Moon C., Kim M.-S., Kim D.-H. (2016). Microbial community analysis of anaerobic granules in phenol-degrading UASB by next generation sequencing. Biochem. Eng. J..

[61] Tsui T.-H., Wu H., Song B., Liu S.-S., Bhardwaj A., Wong J.W.C. (2020). Food waste leachate treatment using an Upflow Anaerobic Sludge Bed (UASB): Effect of conductive material dosage under low and high organic loads. Bioresour. Technol..

[62] Zhao Z., Zhang Y. (2019). Application of ethanol-type fermentation in establishment of direct interspecies electron transfer: A practical engineering case study. Renew. Energy.

[63] Li Y., Chen Y., Wu J. (2019). Enhancement of methane production in anaerobic digestion process: A review. Appl. Energy.

[64] Yang G., Fang H., Wang J., Jia H., Zhang H. (2019). Enhanced anaerobic digestion of Up-Flow Anaerobic Sludge Blanket (UASB) by Blast Furnace Dust (BFD): Feasibility and mechanism. Int. J. Hydrogen Energy.

[65] Srisowmeya G., Chakravarthy M., Nandhini Devi G. (2020). Critical considerations in two-stage anaerobic digestion of food waste—A review. Renew. Sustain. Energy Rev..

[66] Sun C., Liu F., Song Z., Li L., Pan Y., Sheng T., Ren G. (2019). Continuous hydrogen and methane production from the treatment of herbal medicines wastewater in the two-phase ‘UASBH-ICM’ system. Water Sci. Technol..

[67] Rajendran K., Mahapatra D., Venkatraman A.V., Muthuswamy S., Pugazhendhi A. (2020). Advancing anaerobic digestion through two-stage processes: Current developments and future trends. Renew. Sustain. Energy Rev..

[68] Kamyab B., Zilouei H., Rahmanian B. (2019). Investigation of the effect of hydraulic retention time on anaerobic digestion of potato leachate in two-stage mixed-UASB system. Biomass Bioenergy.

[69] Wu J., Jiang B., Feng B., Li L., Moideen S.N.F., Chen H., Mribet C., Li Y.-Y. (2020). Pre-acidification greatly improved granules physicochemical properties and operational stability of Upflow Anaerobic Sludge Blanket (UASB) reactor treating low-strength starch wastewater. Bioresour. Technol..

[70] Diamantis V.I., Kapagiannidis A.G., Ntougias S., Tataki V., Melidis P., Aivasidis A. (2014). Two-stage CSTR–UASB digestion enables superior and alkali addition-free cheese whey treatment. Biochem. Eng. J..

[71] Alpay T., Karabey B., Azbar N., Ozdemir G. (2020). Purified terephthalic acid wastewater treatment using modified two-stage UASB bioreactor systems. Curr. Microbiol..

[72] Jiraprasertwong A., Maitriwong K., Chavadej S. (2019). Production of biogas from cassava wastewater using a three-stage Upflow Anaerobic Sludge Blanket (UASB) reactor. Renew. Energy.

[73] Chavadej S., Wangmor T., Maitriwong K., Chaichirawiwat P., Rangsunvigit P., Intanoo P. (2019). Separate production of hydrogen and methane from cassava wastewater with added cassava residue under a thermophilic temperature in relation to digestibility. J. Biotechnol..

[74] Mainardis M., Flaibani S., Mazzolini F., Peressotti A., Goi D. (2019). Techno-economic analysis of anaerobic digestion implementation in small Italian breweries and evaluation of biochar and granular activated carbon addition effect on methane yield. J. Environ. Chem. Eng..

[75] Siddique N.I., Wahid Z.A. (2018). Achievements and perspectives of anaerobic co-digestion: A review. J. Clean. Prod..

[76] Chan P.C., Lu Q., de Toledo R.A., Gu J.-D., Shim H. (2019). Improved anaerobic co-digestion of food waste and domestic wastewater by copper supplementation—Microbial community change and enhanced effluent quality. Sci. Total Environ..

[77] Loizia P., Neofytou N., Zorpas A.A. (2019). The concept of circular economy strategy in food waste management for the optimization of energy production through anaerobic digestion. Environ. Sci. Pollut. Res..

[78] Kumari K., Suresh S., Arisutha S., Sudhakar K. (2018). Anaerobic co-digestion of different wastes in a UASB reactor. Waste Manag..

[79] Gao M., Zhang L., Liu Y. (2020). High-loading food waste and blackwater anaerobic co-digestion: Maximizing bioenergy recovery. Chem. Eng. J..

[80] Sampaio G.F., Dos Santos A.M., da Costa P.R., Rodriguez R.P., Sancinetti G.P. (2020). High rate of biological removal of sulfate, organic matter, and metals in UASB reactor to treat synthetic acid mine drainage and cheese whey wastewater as carbon source. Water Environ. Res..

[81] Montes J.A., Leivas R., Martínez-Prieto D., Rico C. (2019). Biogas production from the liquid waste of distilled gin production: Optimization of UASB reactor performance with increasing organic loading rate for co-digestion with swine wastewater. Bioresour. Technol..

[82] Rico C., Muñoz N., Fernández J., Rico J.L. (2015). High-load anaerobic co-digestion of cheese whey and liquid fraction of dairy manure in a one-stage UASB process: Limits in co-substrates ratio and organic loading rate. Chem. Eng. J..

[83] Junior A.E.S., Duda R.M., De Oliveira R.A. (2019). Improving the energy balance of ethanol industry with methane production from vinasse and molasses in two-stage anaerobic reactors. J. Clean. Prod..

[84] Ma H., Niu Q., Zhang Y., He S., Li Y.-Y. (2017). Substrate inhibition and concentration control in an UASB-anammox process. Bioresour. Technol..

[85] Zhang F., Li X., Wang Z., Jiang H., Ren S., Peng Y. (2020). Simultaneous ammonium oxidation denitrifying (SAD) in an innovative three-stage process for energy-efficient mature landfill leachate treatment with external sludge reduction. Water Res..

[86] Li X., Lu M., Qiu Q., Huang Y., Li B., Yuan Y., Yuan Y. (2020). the effect of different denitrification and partial nitrification-anammox coupling forms on nitrogen removal from mature landfill leachate at the pilot-scale. Bioresour. Technol..

[87] Li M.-C., Song Y., Shen W., Wang C., Qi W.-K., Peng Y., Li Y.-Y. (2019). The performance of an anaerobic ammonium oxidation upflow anaerobic sludge blanket reactor during natural periodic temperature variations. Bioresour. Technol..

[88] Pekyavas G., Yangin-Gomec C. (2019). Response of anammox bacteria to elevated nitrogen and organic matter in pre-digested chicken waste at a long-term operated UASB reactor initially seeded by methanogenic granules. Bioresour. Technol. Rep..

[89] Mahmod S.S., Azahar A.M., Tan J.P., Jahim J.M., Abdul P.M., Mastar M.S., Anuar N., Mohammed Yunus M.F., Asis A.J., Wu S.-Y. (2019). Operation performance of Up-flow Anaerobic Sludge Blanket (UASB) bioreactor for biohydrogen production by self-granulated sludge using pre-treated Palm Oil Mill Effluent (POME) as carbon source. Renew. Energy.

[90] Moreno Dávila I.M.M., Tamayo Ordoñez M.C., Morales Martínez T.K., Soria Ortiz A.I., Gutiérrez Rodríguez B., Rodríguez de la Garza J.A., Ríos González L.J. (2020). Effect of fermentation time/hydraulic retention time in a UASB reactor for hydrogen production using surface response methodology. Int. J. Hydrogen Energy.

[91] Buitrón G., Muñoz-Páez K.M., Quijano G., Carrillo-Reyes J., Albarrán-Contreras B.A. (2020). Biohydrogen production from winery effluents: Control of the homoacetogenesis through the headspace gas recirculation. J. Chem. Technol. Biotechnol..

[92] Eregowda T., Kokko M.E., Rene E.R., Rintala J., Lens P.N.L. (2020). Volatile fatty acid production from kraft mill foul condensate in upflow anaerobic sludge blanket reactors. Environ. Technol..

[93] Zhang D., Shen J., Shi H., Su G., Jiang X., Li J., Liu X., Mu Y., Wang L. (2019). Substantially enhanced anaerobic reduction of nitrobenzene by biochar stabilized sulfide-modified nanoscale zero-valent iron: Process and mechanisms. Environ. Int..

[94] Chen C., Liang J., Yoza B.A., Li Q.X., Zhan Y., Wang Q. (2017). Evaluation of an Up-Flow Anaerobic Sludge Bed (UASB) reactor containing diatomite and maifanite for the improved treatment of petroleum wastewater. Bioresour. Technol..

[95] El-Khateeb M.A., Emam W.M., Darweesh W.A., El-Sayed E.S.A. (2019). Integration of UASB and down flow hanging non-woven fabric (DHNW) reactors for the treatment of sewage water. Desalin. Water Treat..

[96] Gonzalez-Tineo P.A., Durán-Hinojosa U., Delgadillo-Mirquez L.R., Meza-Escalante E.R., Gortáres-Moroyoqui P., Ulloa-Mercado R.G., Serrano-Palacios D. (2020). Performance improvement of an integrated anaerobic-aerobic hybrid reactor for the treatment of swine wastewater. J. Water Process Eng..

[97] Carvalho J.R.S., Amaral F.M., Florencio L., Kato M.T., Delforno T.P., Gavazza S. (2020). Microaerated UASB reactor treating textile wastewater: The core microbiome and removal of azo dye direct black 22. Chemosphere.

[98] Musa M.A., Idrus S., Harun M.R., Tuan Mohd Marzuki T.F., Abdul Wahab A.M. (2020). A comparative study of biogas production from cattle slaughterhouse wastewater using conventional and modified Upflow Anaerobic Sludge Blanket (UASB) reactors. Int. J. Environ. Res. Public Health.

[99] Wambugu C.W., Rene E.R., van de Vossenberg J., Dupont C., van Hullebusch E.D. (2019). Role of biochar in anaerobic digestion based biorefinery for food waste. Front. Energy Res..

[100] Wu J., Liu Q., Feng B., Kong Z., Jiang B., Li Y.-Y. (2019). Temperature effects on the methanogenesis enhancement and sulfidogenesis suppression in the UASB treatment of sulfate-rich methanol wastewater. Int. Biodeterior. Biodegrad..

[101] Zhang L., Hendrickx T.L.G., Kampman C., Temmink H., Zeeman G. (2013). Co-digestion to support low temperature anaerobic pretreatment of municipal sewage in a UASB–digester. Bioresour. Technol..

[102] Zhang L., De Vrieze J., Hendrickx T.L.G., Wei W., Temmink H., Rijnaarts H., Zeeman G. (2018). Anaerobic treatment of raw domestic wastewater in a UASB-digester at 10 °C and microbial community dynamics. Chem. Eng. J..

[103] McAteer P.G., Christine Trego A., Thorn C., Mahony T., Abram F., O’Flaherty V. (2020). Reactor configuration influences microbial community structure during high-rate, low-temperature anaerobic treatment of dairy wastewater. Bioresour. Technol..

[104] Crone B.C., Garland J.L., Sorial G.A., Vane L.M. (2016). Significance of dissolved methane in effluents of anaerobically treated low strength wastewater and potential for recovery as an energy product: A review. Water Res..

[105] Rongwong W., Goh K., Sethunga G.S.M.D.P., Bae T.-H. (2019). Fouling formation in membrane contactors for methane recovery from anaerobic effluents. J. Membr. Sci..

[106] Hasan M.N., Khan A.A., Ahmad S., Lew B. (2019). Anaerobic and aerobic sewage treatment plants in Northern India: Two years intensive evaluation and perspectives. Environ. Technol. Innov..

[107] Saavedra O., Escalera R., Heredia G., Montoya R., Echeverría I., Villarroel A., Brito L.L. (2019). Evaluation of a domestic wastewater treatment plant at an intermediate city in Cochabamba, Bolivia. Water Pract. Technol..

[108] Lijó L., Malamis S., González-García S., Moreira M.T., Fatone F., Katsou E. (2017). Decentralised schemes for integrated management of wastewater and domestic organic waste: The case of a small community. J. Environ. Manag..

[109] Gao M., Zhang L., Guo B., Zhang Y., Liu Y. (2019). Enhancing biomethane recovery from source-diverted blackwater through hydrogenotrophic methanogenesis dominant pathway. Chem. Eng. J..

[110] Prado L.O., Souza H.H.S., Chiquito G.M., Paulo P.L., Boncz M.A. (2020). A comparison of different scenarios for on-site reuse of blackwater and kitchen waste using the life cycle assessment methodology. Environ. Impact Assess. Rev..

[111] Gao M., Guo B., Zhang L., Zhang Y., Yu N., Liu Y. (2020). Biomethane recovery from source-diverted household blackwater: Impacts from feed sulfate. Process Saf. Environ. Prot..

[112] Slompo N.D.M., Quartaroli L., Zeeman G., da Silva G.H.R., Daniel L.A. (2019). Black water treatment by an Upflow Anaerobic Sludge Blanket (UASB) reactor: A pilot study. Water Sci. Technol..

[113] Gao M., Guo B., Zhang L., Zhang Y., Liu Y. (2019). Microbial community dynamics in anaerobic digesters treating conventional and vacuum toilet flushed blackwater. Water Res..

[114] Adhikari J.R., Lohani S.P. (2019). Design, installation, operation and experimentation of septic tank—UASB wastewater treatment system. Renew. Energy.

[115] Xu S., Zhang L., Huang S., Zeeman G., Rijnaarts H., Liu Y. (2018). Improving the energy efficiency of a pilot-scale UASB-digester for low temperature domestic wastewater treatment. Biochem. Eng. J..

[116] Cunha J.R., Schott C., van der Weijden R.D., Leal L.H., Zeeman G., Buisman C. (2019). Recovery of calcium phosphate granules from black water using a hybrid upflow anaerobic sludge bed and gas-lift reactor. Environ. Res..

[117] Li B., Boiarkina I., Yu W., Huang H.M., Munir T., Wang G.Q., Young B.R. (2019). Phosphorous recovery through struvite crystallization: Challenges for future design. Sci. Total Environ..

[118] Ouhammou B., Aggour M., Frimane Â., Bakraoui M., El Bari H., Essamri A. (2019). A new system design and analysis of a solar bio-digester unit. Energy Convers. Manag..

[119] Kainthola J., Kalamdhad A.S., Goud V.V. (2019). A review on enhanced biogas production from anaerobic digestion of lignocellulosic biomass by different enhancement techniques. Process Biochem..

[120] Rajagopal R., Choudhury M.R., Anwar N., Goyette B., Rahaman M.S. (2019). Influence of pre-hydrolysis on sewage treatment in an Up-Flow Anaerobic Sludge BLANKET (UASB) reactor: A review. Water.

[121] Gurmessa B., Pedretti E.F., Cocco S., Cardelli V., Corti G. (2020). Manure anaerobic digestion effects and the role of pre- and post-treatments on veterinary antibiotics and antibiotic resistance genes removal efficiency. Sci. Total Environ..

[122] Domínguez-Maldonado J.A., Alzate-Gaviria L., Milquez-Sanabria H.A., Tapia-Tussell R., Leal-Bautista R.M., España-Gamboa E.I. (2019). Chemical pretreatments to enrich the acidogenic phase in a system coupled packed bed reactor with a UASB reactor using peels and rotten onion waste. Waste Biomass Valorization.

[123] Paulista L.O., Boaventura R.A.R., Vilar V.J.P., Pinheiro A.L.N., Martins R.J.E. (2020). Enhancing methane yield from crude glycerol anaerobic digestion by coupling with ultrasound or A. Niger/E. Coli biodegradation. Environ. Sci. Pollut. Res..

[124] Eftaxias A., Diamantis V., Michailidis C., Stamatelatou K., Aivasidis A. (2020). The role of emulsification as pre-treatment on the anaerobic digestion of oleic acid: Process performance, modeling, and sludge metabolic properties. Biomass Convers Biorefinery.

[125] Uddin M.N., Rahman M.A., Taweekun J., Techato K., Mofijur M., Rasul M. (2019). Enhancement of biogas generation in Up-Flow Sludge Blanket (UASB) bioreactor from Palm Oil Mill Effluent (POME). Energy Procedia.

[126] Zhang L. (2020). Advanced treatment of oilfield wastewater by a combination of DAF, yeast bioreactor, UASB, and BAF processes. Sep. Sci. Technol..

[127] Gadow S.I., Li Y.-Y. (2020). Development of an integrated anaerobic/aerobic bioreactor for biodegradation of recalcitrant azo dye and bioenergy recovery: HRT effects and functional resilience. Bioresour. Technol. Rep..

[128] Owaes M., Gaur R.Z., Hasan M.N., Gani K.M., Kumari S., Bux F., Khan A.A., Kazmi A. (2020). Performance assessment of aerobic granulation for the post treatment of anaerobic effluents. Environ. Technol. Innov..

[129] Leite L.d.S., Hoffmann M.T., Daniel L.A. (2019). Microalgae cultivation for municipal and piggery wastewater treatment in Brazil. J. Water Process Eng..

[130] Walia R., Kumar P., Mehrotra I. (2020). Post-treatment of effluent from UASB reactor by surface aerator. Int. J. Environ. Sci. Technol..

[131] Tarpani R.R.Z., Alfonsín C., Hospido A., Azapagic A. (2020). Life cycle environmental impacts of sewage sludge treatment methods for resource recovery considering ecotoxicity of heavy metals and pharmaceutical and personal care products. J. Environ. Manag..

[132] Kumar M., Gogoi A., Mukherjee S. (2020). Metal removal, partitioning and phase distributions in the wastewater and sludge: Performance evaluation of conventional, upflow anaerobic sludge blanket and downflow hanging sponge treatment systems. J. Clean. Prod..

[133] de Souza Celente G., Colares G.S., da Silva Araújo P., Machado Ê.L., Lobo E.A. (2020). Acute ecotoxicity and genotoxicity assessment of two wastewater treatment units. Environ. Sci. Pollut. Res. Int..

[134] Braga A.F.M., Zaiat M., Silva G.H.R., Fermoso F.G. (2017). Metal fractionation in sludge from sewage UASB treatment. J. Environ. Manag..

[135] Zeng T., Rene E.R., Hu Q., Lens P.N.L. (2019). Continuous biological removal of selenate in the presence of cadmium and zinc in UASB reactors at psychrophilic and mesophilic conditions. Biochem. Eng. J..

[136] Hou J., Chen Z., Gao J., Xie Y., Li L., Qin S., Wang Q., Mao D., Luo Y. (2019). Simultaneous removal of antibiotics and antibiotic resistance genes from pharmaceutical wastewater using the combinations of up-flow anaerobic sludge bed, anoxic-oxic tank, and advanced oxidation technologies. Water Res..

[137] Mainardis M., Buttazzoni M., De Bortoli N., Mion M., Goi D. (2020). Evaluation of ozonation applicability to pulp and paper streams for a sustainable wastewater treatment. J. Clean. Prod..

[138] Qian M., Yang L., Chen X., Li K., Xue W., Li Y., Zhao H., Cao G., Guan X., Shen G. (2020). The treatment of veterinary antibiotics in swine wastewater by biodegradation and fenton-like oxidation. Sci. Total Environ..

[139] Freitas F.F., De Souza S.S., Ferreira L.R.A., Otto R.B., Alessio F.J., De Souza S.N.M., Venturini O.J., Ando Junior O.H. (2019). The Brazilian market of distributed biogas generation: Overview, technological development and case study. Renew. Sustain. Energy Rev..

[140] Meneses-Jácome A., Diaz-Chavez R., Velásquez-Arredondo H.I., Cárdenas-Chávez D.L., Parra R., Ruiz-Colorado A.A. (2016). Sustainable energy from agro-industrial wastewaters in Latin-America. Renew. Sustain. Energy Rev..

[141] Bressani-Ribeiro T., Chamhum-Silva L.A., Chernicharo C.A.L. (2019). Constraints, performance and perspectives of anaerobic sewage treatment: Lessons from full-scale sewage treatment plants in Brazil. Water Sci. Technol..

[142] Gaur R.Z., Khan A.A., Lew B., Diamantis V., Kazmi A.A. (2017). Performance of full-scale UASB reactors treating low or medium strength municipal wastewater. Environ. Process..

[143] Lopes T.A.S., Queiroz L.M., Torres E.A., Kiperstok A. (2020). Low complexity wastewater treatment process in developing countries: A LCA approach to evaluate environmental gains. Sci. Total Environ..

[144] Maharjan N., Nomoto N., Tagawa T., Okubo T., Uemura S., Khalil N., Hatamoto M., Yamaguchi T., Harada H. (2019). Assessment of UASB-DHS technology for sewage treatment: A comparative study from a sustainability perspective. Environ. Technol..

[145] Rosa A.P., Chernicharo C.A.L., Lobato L.C.S., Silva R.V., Padilha R.F., Borges J.M. (2018). Assessing the potential of renewable energy sources (biogas and sludge) in a full-scale UASB-based treatment plant. Renew. Energy.

[146] Oliveira J.F.d., Fia R., Fia F.R.L., Rodrigues F.N., Matos M.P.d., Siniscalchi L.A.B. (2020). Principal component analysis as a criterion for monitoring variable organic load of swine wastewater in integrated biological reactors UASB, SABF and HSSF-CW. J. Environ. Manag..

